# The Role of Mean Platelet Volume in the Diagnosis of Acute Appendicitis: A Retrospective Case-Controlled Study

**DOI:** 10.5812/ircmj.11934

**Published:** 2013-12-05

**Authors:** Huseyin Narci, Emin Turk, Erdal Karagulle, Turhan Togan, Keziban Karabulut

**Affiliations:** 1 Department of Emergency Medicine, Baskent University Faculty of Medicine, Ankara, Turkey; 2 Department of General Surgery, Baskent University Faculty of Medicine, Ankara, Turkey; 3 Department of Infectious Diseases and Clinical Microbiology, Baskent University Faculty of Medicine, Ankara, Turkey

**Keywords:** Appendicitis, C-Reactive Protein, Leukocytes, Blood Platelets

## Abstract

**Background:**

The level of platelet volume (MPV) has been reported to be a laboratory marker in inflammatory cases.

**Objectives:**

The aim of this study was to seek whether MPV has a role in the diagnosis of acute appendicitis. It was also aimed to show the relationship of MPV with leukocyte count and C-reactive protein (CRP) level.

**Materials and Methods:**

This study was conducted via retrospective assessment of the hospital records of the adult patients who were operated for acute appendicitis between January 2010 and December 2012 and had a pathology report that confirmed the diagnosis of acute appendicitis. The patients in the control group were selected from healthy adults of similar age who applied to check-up clinic. The number of essential cases was defined by performing power analysis. Age, gender, leukocyte count, CRP, and MPV values were recorded. This study is a case controlled retrospective clinical study.

**Results:**

A total of 503 patients in the acute appendicitis group and 121 patients in the control group were included, making up a total of 624 subjects. The median MPV levels were 7.92 ± 1.68 fL in the acute appendicitis group, while 7.43 ± 1.34 fL in the control group. CRP, leukocyte count, and MPV level were significantly higher in the acute appendicitis group (P < 0.001). MPV, leukocyte count, and CRP had a sensitivity and specificity of 66% and 51%; 91% and 74%; and 97% and 41%, respectively. No correlation was found between MPV, CRP, and leukocyte count.

**Conclusions:**

MPV level was higher in patients with acute appendicitis. MPV may guide the diagnostic process of acute appendicitis. However, we detected that the sensitivity and specificity of leukocyte count and CRP were superior to those of MPV in the diagnosis of acute appendicitis.

## 1. Background

Acute appendicitis is a common surgical condition of the abdomen, the prompt diagnosis of which is rewarded by a marked decrease in morbidity and mortality (1). Classically, the diagnosis of acute appendicitis is based on a brief history of abdominal pain, nausea, migration of pain to the right iliac fossa, and signs of local peritonitis; diagnostic accuracy based on these symptoms ranges from 70% to 80% (1, 2). Therefore, diagnostic errors are common, resulting in a median incidence of perforation of 20% and a negative laparotomy rate ranging from 2% to 30% (1).

The preoperative laboratory tests can be performed easily in primary healthcare settings and often aid primary clinicians with decision making about patients with clinically suspected acute appendicitis. Several parameters (i.e. C-reactive protein (CRP), white blood cell count, lymphocyte/leukocyte rate, interleukin-6, interleukin-10, interleukin-4, interleukin-5, interleukin-12, tumor necrosis factor alpha, endotoxin, erythrocyte sedimentation rate, procalcitonin, fibrinogen, alpha 2 - macroglobulin, alpha 1-antitrypsin, D-Lactate) for the diagnosis of acute appendicitis have been investigated in the literature (2). Mean platelet volume (MPV) is a measure of platelet size, generated by full blood count analyzers as part of the routine complete blood count test cycle which is commonly overlooked by clinicians (3). MPV is one of the most widely used surrogate markers of platelet function and has been shown to reflect inflammatory burden and disease activity in several diseases including pre-eclampsia, acute pancreatitis, unstable angina, myocardial infarction, and systemic inflammation such as ulcerative colitis and Crohn’s disease (4).

## 2. Objectives

There are only a few studies reporting that MPV levels may be valuable in the diagnosis of acute appendicitis and the results are controversial (5-7). In the present study we aimed to seek whether MPV level is important in the diagnosis of acute appendicitis. In addition, it was aimed to show the relationship of MPV level with leukocyte count and CRP level.

## 3. Materials and Method

The main analysis in this study was the comparison of the difference MPV measurements between acute appendicitis and control groups. In healthy individuals MPV levels have been reported as 7 to 12 fL with a standard deviation of approximately 1.2 fL. A 0.5 fL difference in the mean MPV values was determined to represent a significant difference between acute appendicitis and control groups. Given these assumptions and assuming that two-sample independent t test was to be used for comparison of means, it was determined that both groups had to include at least 123 test subjects to achieve a power level of 90%. Sample sizes were calculated by using the Minitab statistical package software (Release 14). This study was conducted via retrospective assessment of hospital records of the adult patients who were operated for acute appendicitis in Baskent University Konya Research and Application Center (Konya / Turkey) between January 2010 and December 2012. This study was approved by Baskent University Institutional Review Board and supported by Baskent University Research Fund (KA 10 / 50). “Inclusion criteria involved that all patients were adults, and their pathology reports confirmed the diagnosis of acute appendicitis. The exclusion criteria in the acute appendicitis group included heart failure, hematological disease, cancer, chronic infections, hepatic disease, and vascular disease. A total of 570 patient files were included in the acute appendicitis group. Sixty-seven of them were excluded by the exclusion criterias and 503 patients included the study. This patients file inclusion/exclusion process was made by a surgeon (ET). The patients in the control group were selected from healthy adults of similar age who applied to check-up clinic and had no active complaint, chronic disease, or abnormal physical examination. Age, gender, leukocyte count, and CRP and MPV levels were recorded. This study is a case controlled retrospective clinical study.

### 3.1. Laboratory Measurements

WBC counts were determined using an electronic cell counter (Cell-Dyne 3700, Abbott, Abbott Park, IL, USA). Serum CRP levels were measured by spectrophotometric methods (Abbott Aeroset, Tokyo, Japan). The expected MPV values in our laboratory ranged between 7.0 and 12 fL.

### 3.2. Statistical Analysis

Statistical analyses were performed with SPSS software (SPSS: An IBM Company, version 9.0, IBM Corporation, and Armonk, New York, USA). The groups were compared using the t test for continuous variables and chi-square test for categorical variables. The measurements of MPV, leukocytes and CRP were not normally distributed. Therefore, non-parametric statistical analysis was used”. Mann-Whitney U test was used to compare nonhomogenous groups in pairs. A simple correlation test (Spearman’s test) was used to observe the correlation between the MPV and other variables. Numeric values were expressed as means (SD). A P value less than .05 was considered statistically significant. ROC curve analysis method was used to determine the relation between measurments (MPV, CRP and leukocytes) and acute appendisitis condition.

## 4. Results

**Figure 1.**
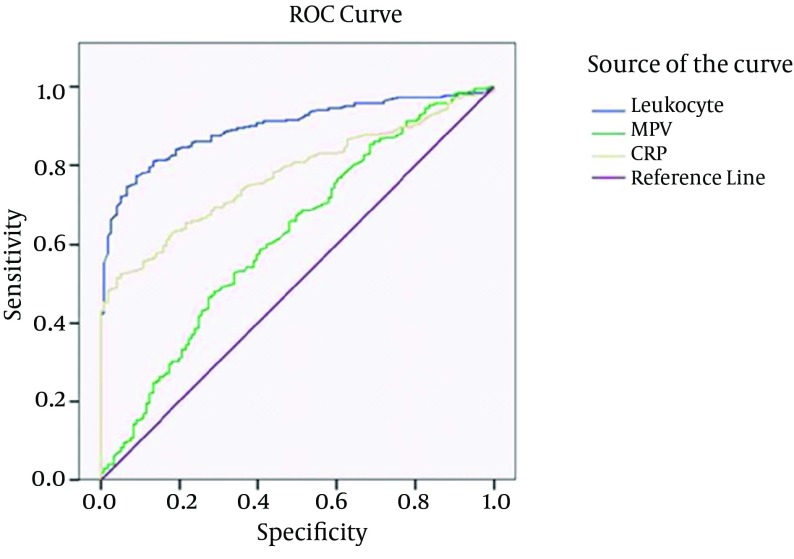
Receiver Operating Characteristic (ROC) Curve of Mean platelet Volume (MPV), Leukocyte, and C-Reactive Protein (CRP)

A total of 503 patients were included in the acute appendicitis group and 121 patients were included in the control group, making up a total of 624 subjects. No significant difference was observed between the acute appendicitis and control groups with respect to age and gender P > 0.05 (Table 1). The median leukocyte count was 13500 ± 5500 mm3 in the acute appendicitis group and 7270 ± 2590 mm3 in the control group. The leukocyte count was significantly higher in the acute appendicitis group (P < 0.001). The median CRP level was 15.26 ± 58.55 mg/dL in the acute appendicitis group and 2.6 ± 5.09 mg/dL in the control group. CRP level in the acute appendicitis group was significantly higher compared with the control group (P < 0.001). The median MPV level was 7.92 ± 1.68 fL in the acute appendicitis group and 7.43 ± 1.34 fL in the control group. MPV level was significantly higher in the acute appendicitis group compared with the control group. (P < 0.001) (Table 1). Receiver operating characteristic curve analysis suggested that the best cutoff point for MPV in the diagnosis of acute appendicitis was 7.87 fL, which had a sensitivity of 66% and a specificity of 51%, (area under curve [AUC]: 0,62; Figure 1). Receiver operating characteristic curve analysis suggested that the best cutoff point for leukocyte count in the diagnosis of acute appendicitis was 10450/mm ^3 ^, which had a sensitivity of 91% and a specificity of 74% (area under curve [AUC]: 0.9; Figure 1). Receiver operating characteristic curve analysis suggested the best cutoff point for CRP level in the diagnosis of acute appendicitis was 27.1 mg/dL, which had a sensitivity of 97% and a specificity of 41% (area under curve [AUC]: 0.77; Figure 1). No correlation was found between MPV, CRP, and leukocyte levels (Table 2). 

**Table 1.** Comparison of the demographic features and leukocyte count, CRP, and MPV levels of the subjects in the acute appendicitis and the control groups.

	Acute Appendicitis (n=503)	Control Group (n=121 )	P-Value
**Male / female**	283/220	69/52	0.85
**Age (y) **	34.7 (14.1)	35.2 (8.1)	0.71
**Leukocyte (**mm^3^**) **	13500 (5500)	7270 (2590)	< 0.001
**CRP (mg/L) ** ^**a**^	15.26 (58.55)	2.6 (5.09)	< 0.001
**MPV(fL) ** ^**a**^	7.92 (1.68)	7.43 ( 1.34)	< 0.001

^a^Abbreviations: CRP: C-reactive protein; MPV: mean platelet volume

**Table 2.** Correlation Analysis of Leukocyte, CRP, and MPV Levels in Patients with Acute Appendicitis.

Parameters	Correlation Coefficient (r)	P value
**Leukocyte - MPV**	0.031	0.49
**Leukocyte - CRP**	-0.03	0.51
**CRP – MPV**	0.006	0.9

## 5. Discussion

The pathophysiology of acute appendicitis is characterized by the mucosal ischemia of the appendix that results from ongoing mucus secretion from the appendiceal mucosa distal to an obstruction of the lumen, elevating intraluminal and, in turn, venous pressures. Once luminal pressure exceeds 85 mmHg, venules that drain the appendix become thrombosed and, in the setting of continued arteriolar in flow, vascular congestion and engorgement of the appendix become manifest (2). Infection is added to the inflammation of appendicitis.

WBC count is most frequently used to laboratory test for diagnosis acute appendicitis. Several reports have suggested that an elevated WBC count is usually the earliest laboratory measure to indicate inflammation of the appendix, and most patients with acute appendicitis present with leukocytosis (1, 8). We found that WBC count was significantly higher in acute appendicitis. In various studies, the range of sensitivity and specificity of WBC in the diagnosis of acute appendicitis have been reported 67 - 97.8% and 31.9 -80%, respectively (5). Similar to the literature, the present study found that the sensitivity and specificity of leukocyte level were 91 and 74%, respectively.

CRP is a sensitive acute phase protein that lacks specificity due to increased levels in all acute inflammatory processes. Its concentration increases with the duration and extent of the inflammation. In a meta-analysis examining the accuracy of CRP levels in the diagnosis of acute appendicitis, a wide range of sensitivity (40 – 99%) and specificity (27 - 90%) was found in literature (9). Similar to the literature, this study found a sensitivity of 97% and a specificity of 41% for CRP in the diagnosis of acute appendicitis. Among the assessed parameters, CRP had the highest sensitivity and the lowest specificity.

MPV is a simple and accurate marker of the functional status of platelets. Higher MPV values usually reflect augmented production of young platelets and increased number of large hyperaggregable platelets. Thus, MPV has been considered a suitable indicator of platelet activation (10, 11). As described previously, larger platelets are more reactive. Platelet size is determined at the level of the progenitor cell (i.e. the megakaryocyte), and studies have reported that cytokines, such as interleukin-3 or interleukin-6, influence megakaryocyte ploidy and can lead to the production of more reactive, larger platelets (4-7) Thus, platelet volume has been proposed as an indirect marker of increased platelet reactivity. Additionally, activated platelets release antibacterial peptides (12); however, some evidence indicates that certain pathogens may have developed a means to exploit activated platelets by binding to their surfaces to establish or propagate infection (13). In addition, previous studies have reported an association between changes in the levels of MPV and various non-infectious inflammatory processes, which may suggest that MPV changes reflect disease activity in inflammation (14-16).

Albayrak et al. (5) in a 226-patient study, found a significantly lower MPV level in patients with acute appendicitis compared to the control group. They suggested that MPV level may guide the management of patients suspected to have acute appendicitis (5). They found a cutoff level of below 7.6 fL for MPV. We found a cutoff level of above 7.87 fL in patients with acute appendicitis. In a pediatric age group, Bilici et al. (6) found that the MPV level significantly decreased in acute appendicitis compared with the control group (6). Uyanik et al. (7) on the other hand, reported that the MPV level was not predictive in the diagnosis of acute appendicitis in pediatric patients (7). Unlike these studies, we found a significantly higher MPV level in the acute appendicitis group than the control group. In contrast, Albayrak et al. and Bilici et al. reported lower MPV levels in the appendicitis group compared to controls. Those studies reported a sensitivity level of 73 and 87%, and a specificity level of 84 and 54%, respectively. In our study MPV's sensitivity was 66%, a value lower than previously reported ones, and its specificity was 51%. Based on these findings, it can be suggested that leukocyte count and CRP levels may be superior to MPV in diagnosis acute appendicitis.

In conclusion, MPV level was higher in patients with acute appendicitis compared to the control group in a retrospective case-controlled our study. However, literature data on this subject are controversial and obtained from limited patient numbers. Our study is the largest study performed so far in terms of patient number. MPV may guide the diagnostic process of acute appendicitis. However, our study revealed that the sensitivity and specificity of leukocyte count and CRP were superior to those of MPV in diagnosis of acute appendicitis. We think that further prospective, multicenter studies with a large sample size are needed in this field.
